# Deep learning for emergency ascites diagnosis using ultrasonography images

**DOI:** 10.1002/acm2.13695

**Published:** 2022-06-20

**Authors:** Zhanye Lin, Zhengyi Li, Peng Cao, Yingying Lin, Fengting Liang, Jiajun He, Libing Huang

**Affiliations:** ^1^ Shantou University Medical College Shantou China; ^2^ Department of Ultrasound The First Affiliated Hospital of Shenzhen University Shenzhen Second People's Hospital Shenzhen China; ^3^ Department of Diagnostic Radiology The University of Hong Kong Hong Kong China; ^4^ South China University of Technology Guangzhou China

**Keywords:** abdominal free fluid, deep learning, emergency, ultrasonography

## Abstract

**Purpose:**

The detection of abdominal free fluid or hemoperitoneum can provide critical information for clinical diagnosis and treatment, particularly in emergencies. This study investigates the use of deep learning (DL) for identifying peritoneal free fluid in ultrasonography (US) images of the abdominal cavity, which can help inexperienced physicians or non‐professional people in diagnosis. It focuses specifically on first‐response scenarios involving focused assessment with sonography for trauma (FAST) technique.

**Methods:**

A total of 2985 US images were collected from ascites patients treated from 1 January 2016 to 31 December 2017 at the Shenzhen Second People's Hospital. The data were categorized as Ascites‐1, Ascites‐2, or Ascites‐3, based on the surrounding anatomy. A uniform standard for regions of interest (ROIs) and the lack of obstruction from acoustic shadow was used to classify positive samples. These images were then divided into training (90%) and test (10%) datasets to evaluate the performance of a U‐net model, utilizing an encoder–decoder architecture and contracting and expansive paths, developed as part of the study.

**Results:**

Test results produced sensitivity and specificity values of 94.38% and 68.13%, respectively, in the diagnosis of Ascites‐1 US images, with an average Dice coefficient of 0.65 (standard deviation [SD] = 0.21). Similarly, the sensitivity and specificity for Ascites‐2 were 97.12% and 86.33%, respectively, with an average Dice coefficient of 0.79 (SD = 0.14). The accuracy and area under the curve (AUC) were 81.25% and 0.76 for Ascites‐1 and 91.73% and 0.91 for Ascites‐2.

**Conclusion:**

The results produced by the U‐net demonstrate the viability of DL for automated ascites diagnosis. This suggests the proposed technique could be highly valuable for improving FAST‐based preliminary diagnoses, particularly in emergency scenarios.

## 1 INTRODUCTION

Abdominal trauma is a common injury^1^, ^2^, ^3^, ^4^, ^5^, ^6^, ^7^, ^8^, ^9^, ^10^ potentially leading to active bleeding, caused by liver or spleen damage, which is the leading cause of death after trauma.^11^ Patients suffering from intra‐abdominal injuries can be divided into hemodynamically stable and unstable categories.^10^ Those with obvious signs of hemodynamic instability require active intervention and often a laparotomy. In contrast, a variety of alternative examination methods are available for hemodynamically stable individuals.^12^ The current clinical consensus is that hemodynamic stability is the only determining factor for non‐surgical treatment. However, approximately 10% of such patients undergo surgery during treatment, often out of necessity.^13^

As such, the ability to produce an accurate diagnosis quickly, in order to make accurate decisions concerning the need for surgery, would be of significant clinical benefit. For example, some blunt abdominal trauma patients require emergency surgical consultation and a laparotomy through physical examination, including cases of peritonitis or open pelvic fractures. However, abdominal injuries (liver or spleen rupture, gastrointestinal perforations, etc.) are often difficult to diagnose with a physical examination and clinical signs typically do not provide sufficient information concerning the need for an operation. In addition, it is possible for patients exhibiting normal physical examinations and vital signs to be suffering from abdominal injuries.^12^, ^14^, ^15^, ^16^, ^17^ As a result, the assessment of abdominal trauma, particularly blunt abdominal trauma, remains challenging.

Early image‐based examinations are critical for trauma identification. For instance, mortality increases by approximately 1% for every 3 min of treatment delay for patients who require a laparotomy. Focused assessment with sonography for trauma (FAST) is a non‐invasive examination technique that has been widely used in the detection of abdominal trauma.^18^ Specific areas in the abdomen are often examined for the presence of abdominal free fluid, a strong indication of severe intra‐abdominal injuries that may require an emergency laparotomy.^16^ Several studies have shown that FAST can guide clinical decision‐making and determine the need for angiography or surgery, particularly for children, pregnant women, and patients exhibiting hemodynamic instabilities.^3^, ^10^, ^17^, ^19^, ^20^, ^21^, ^22^, ^23^, ^24^, ^25^ FAST can be performed at the bedside in 3‐4 min, repeatedly if need be, and avoids the risks associated with transporting patients and radiation.^3^, ^14^, ^26^ FAST identifies the presence of free fluid in the abdominal cavity, which is typically thought to be secondary to serious abdominal trauma.^27^ In the supine position, free fluid typically accumulates in specific areas like the hepatorenal fossa. Peritoneal free fluid detected by FAST may also provide information for clinical diagnosis and treatment in patients with stable conditions, such as the need for blood products.^16^, ^28^, ^29^, ^30^, ^31^ As such, the early detection of abdominal free fluid is critical for the treatment of trauma patients in a variety of situations.

The use of artificial intelligence (AI) for medical applications has been expanding across multiple fields in recent years and deep learning (DL), specifically, has become one of the most popular computer‐aided diagnosis (CAD) techniques for ultrasonography (US) images.^32^, ^33^, ^34^, ^35^, ^36^, ^37^, ^38^ DL has previously been applied to the CAD of ascites.^21^ It is a data‐driven methodology that can be used to extract and learn nonlinear features from data, without requiring domain expertise.^39^ This is particularly beneficial for first‐response and emergency situations, where access to specialists is limited. The U‐net is a DL model with an encoder–decoder architecture that was first proposed in 2015.^40^, ^41^, ^42^ U‐nets have previously been applied to biomedical image processing problems such as liver and tumor segmentation in computed tomography (CT) scans, mass and calcification detection in digital mammograms, and the segmentation of skin lesions.^40^, ^42^, ^43^, ^44^, ^45^ These models are composed of multiple layers and thus learn different hierarchical features in each iteration.^46^ Scholars have recently improved on the basic U‐net structure, proposing more powerful frameworks such as UNet++, UNET 3+, and H‐DenseUNet.^41^, ^43^, ^47^

It is not difficult for a physician with experience in ultrasound to identify peritoneal free fluid in US images. However, the identification of abdominal free fluid can still be time consuming for novice physicians, clinicians without ultrasonic imaging expertise, or non‐professional people. Thus, the proposed technique could be used to rapidly identify and locate peritoneal free fluid, thereby reducing examination times and leading to faster intervention. In addition, portable ultrasound is becoming more widely available and AI could enable those without a diagnostic background, such as emergency medical technicians, to use ultrasound in making decisions. A system of this type could also help novice physicians to learn and progress. As such, the primary objective of this study is to determine the viability of deep learning algorithms for timely CAD of abdominal free fluid in US images collected using FAST.

## 2 MATERIALS AND METHODS

A total of 2985 US images were collected from 845 ascites patients (45.43 ± 20.68 years old) from 1 January 2016 to 31 December 2017 as part of the ultrasonic picture archiving and communication systems (PACS) of the Shenzhen Second People's Hospital. The intended application environment for our study is primarily emergency and teaching situations. As such, we do not assume uniform ultrasonic equipment, interfacing software, scanning parameters, and so forth. In other words, the images we collected come from multivendor equipment, different doctors, and different scanning parameters. Ascites image classification was conducted using a predefined standard that can be described as follows. (1) The images must clearly exhibit abdominal free fluid (the affected region must be visible in the images). (2) Images containing the liver or spleen are classified as Ascites‐1. Images in which the liver, spleen, uterus, or bladder cannot be seen are classified as Ascites‐2. Images containing the uterus or bladder are classified as Ascites‐3 (not included as part of the study). Images were anonymized and all personal information from patients was removed during processing. As a result, the need for informed consent was waived.

**FIGURE 1:**
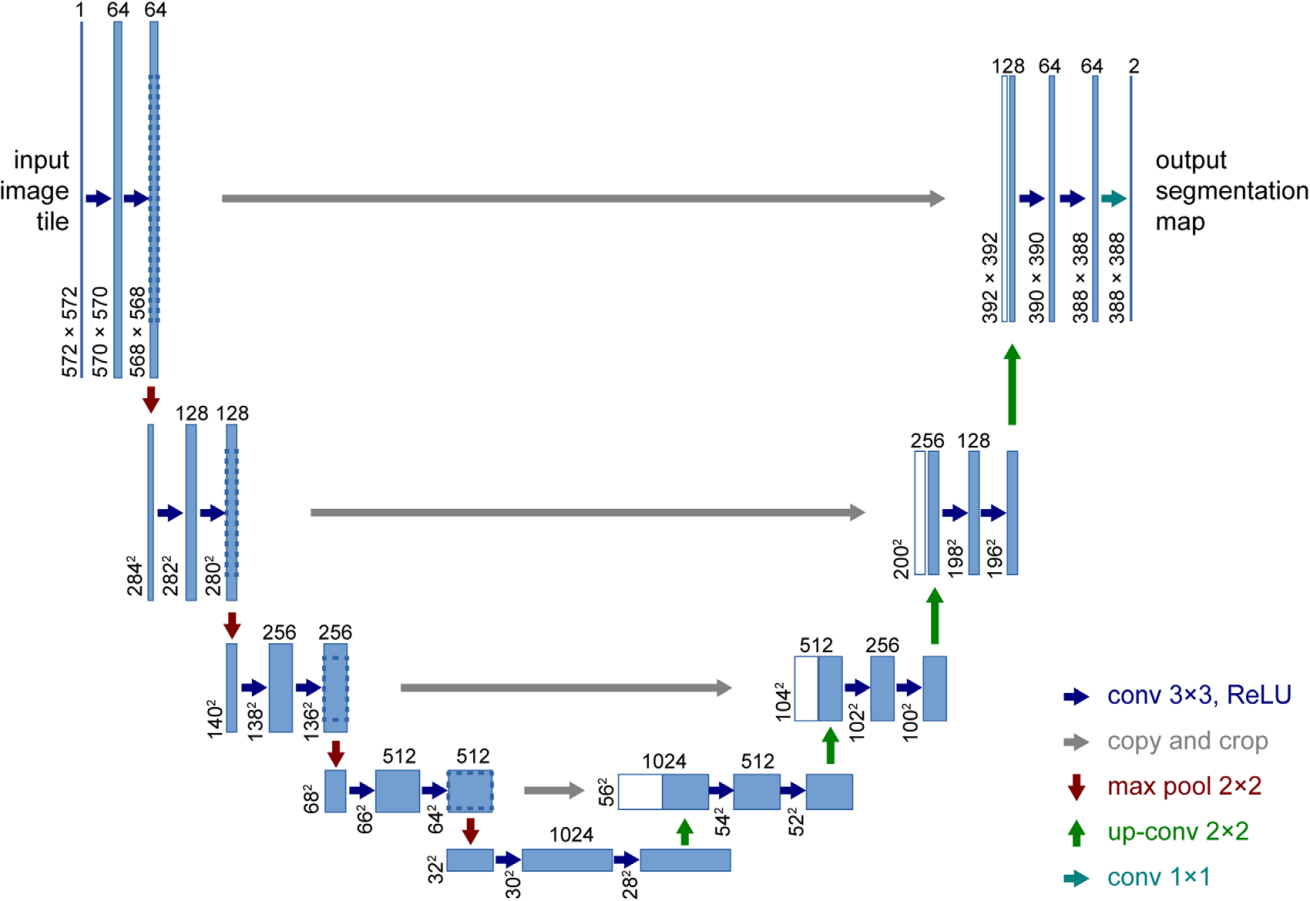
The U‐net architecture with example for 32 × 32 pixels. Reprinted with permission from Springer Nature Customer Service Center GmbH^42^

A U‐net model exhibiting contracting and expansive paths, often the basis of biomedical image segmentation networks, was developed as part of the study. The contracting path involved a repeated application of two 3 × 3 convolutions, each followed by a rectified linear unit (ReLU). The contracting path also included one max‐pooling layer utilizing a 2 × 2 kernel. The expansive path consisted of a repeated concatenation of features extracted from corresponding layers in the contracting path, two convolution layers, and one upsampling layer (see Figure 1). The 2 × 2 convolution layer kernel was the same as that of the contracting path and upsampling layer. The U‐net included 23 total convolutional layers, the last of which exhibited a 1 × 1 convolution kernel, and was trained over 200 epochs with a batch size of 16.^40^, ^42^ A binary cross‐entropy loss function was applied with a learning rate of 0.0001, an Adam optimizer, and a He‐normal weight initializer function.^40^, ^48^, ^49^ All experiments were performed on an Intel Core i5 (12G) computer with a single GeForce GTX 1080 Ti GPU. Python (version 3.6) running on a Windows 10 operating system was used to process the images.

**FIGURE 2:**
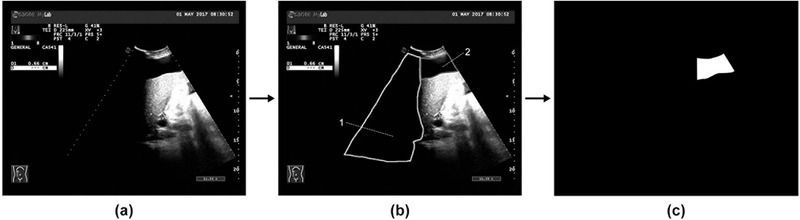
(a) A sample image collected from picture archiving and communication systems (PACS). (b) Area‐2 denotes regions of interest (ROIs) while Area‐1 indicates an obstruction from acoustic shadows. (c) The image after annotation. Pictures (a) and (c) were input to U‐net

Supervised learning was implemented to simplify the training and test process.^50^, ^51^ The images need to be annotated (pre‐processed). Areas exhibiting clear free fluid in positive images were manually delineated and denoted as regions of interest (ROIs), excluding those that could not be identified due to the presence of acoustic shadows (see Figure 2). The detection of ROIs and the annotation process are done by an experienced physician.

**FIGURE 3:**
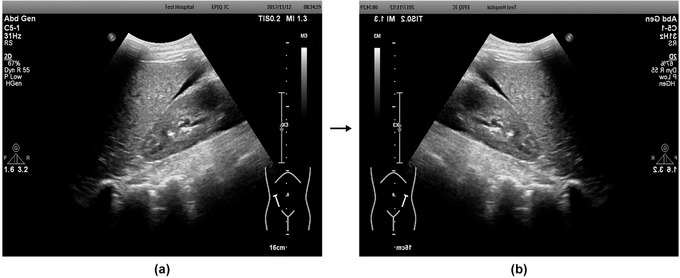
(a) An image prior to horizontal mirroring. (b) The image after horizontal mirroring (data augmentation)

The demand for large quantities of annotated data is an obstacle to the development of robust AI systems.^52^ Data augmentation is a better solution. Data augmentation can be used to reduce overfitting and improve performance. In this process, new data (images) are produced by manipulating the original data with strategies such as flipping, rotation, translation, and noise injection.^42^, ^52^, ^53^, ^54^, ^55^ In our study, we use the strategy of horizontally flipping the images. Horizontally flipping retained the boundaries, echo details, and other important structural features, since the fluid always produces posterior acoustic enhancement effects in US images (see Figure 3). GNU Image Manipulation Program (GIMP, version 2.10.22) and Adobe Photoshop CS6 software were used to process the annotated results. The number of images reached 5970 by the data augmentation step.

After data augmentation, positive and negative ascites images were randomly assigned to training and test sets. The proportion of the training set and the test set was about 90/10. External validation, with data not involved in model development (here it refers to the previously mentioned test set), was used to assess U‐net performance and reduce overestimation of diagnostic accuracy.^56^ Receiver operating characteristic (ROC) curve, sensitivity, specificity, accuracy, area under the curve (AUC), and average Dice coefficient were included as evaluation metrics. The Dice coefficient is a measure of contour consistency for segmented lesions occupying the same position and can be calculated as:

$$\begin{array}{c} Dice\;=\frac{2\left|X\cap Y\right|}{\left|X\right|+\left|Y\right|}\;\#\left(1\right) \end{array}$$

where *|X|* and *|Y*| indicate the number of voxels in segmentations *X* and *Y*, and *X ∩ Y* defines the set of voxels that overlap between segmentations *X* and *Y*.^57^, ^58^ The ascites images after annotation are regarded as a reference and compared with the results of U‐net.

## 3 PROCESSES AND RESULTS

In the first test, 900 US images exhibiting peritoneal free fluid (including Ascites‐1, Ascites‐2, and Ascites‐3) were selected to form the training set, while 182 images comprised the test set. The program achieved an average Dice coefficient of 0.61 (standard deviation [SD] = 0.26). While this value is relatively low, the location and segmentation of ROIs were secondary to diagnosing the presence of ascites, which was the primary goal of the study. Testing was repeated after grouping to compensate for significant differences in US images exhibiting peritoneal effusion in different areas. Model training was conducted using 627 Ascites‐1, 640 Ascites‐2, and 422 Ascites‐3 images. Testing involved 71 Ascites‐1, 72 Ascites‐2, and 47 Ascites‐3 images. Test results produced average Dice coefficients of 0.51 (SD = 0.26), 0.76 (SD = 0.17), and 0.60 (SD = 0.25) for Ascites‐1, Ascites‐2, and Ascites‐3, respectively. Low correctness was observed in the segmentation of Ascites‐1 and Ascites‐3, which suggests the U‐net is ideally suited to Ascites‐2.

**FIGURE 4:**
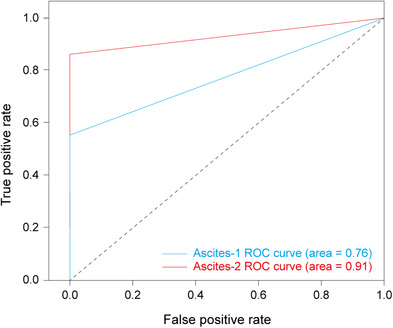
A receiver operating characteristic (ROC) curve for Ascites‐1 and Ascites‐2

Error was reduced by uniformly investigating the ROIs of all positive images, re‐annotating according to a predefined standard. Data augmentation was done at this time. The U‐net was then further trained using 2872 Ascites‐1 images (1436 positive and 1436 negative) and 2500 Ascites‐2 images (1250 positive and 1250 negative). The corresponding test set was composed of 320 Ascites‐1 images (160 positive and 160 negative) and 278 Ascites‐2 images (139 positive and 139 negative). Diagnostic sensitivity and specificity for Ascites‐1 were 94.38% and 68.13%, respectively, with an average Dice coefficient of 0.65 (SD = 0.21). The sensitivity and specificity for Ascites‐2 were 97.12% and 86.33%, respectively, with an average Dice coefficient of 0.79 (SD = 0.14). The accuracy was 81.25% for Ascites‐1 and 91.73% for Ascites‐2 (see Tables 1 and 2). The AUC was 0.76 for Ascites‐1 and 0.91 for Ascites‐2 (see Figure 4).

**TABLE 1:** Numbers for training and test images used in the final training and test

	**Ascites‐1**		**Ascites‐2**		
	Training set	Test set	Training set	Test set	**Total**
Positive	1436	160	1250	139	2985
Negative	1436	160	1250	139	2985
All	2872	320	2500	278	5970

**TABLE 2:** Results of the final test

	**Evaluation metrics**						
	Sensitivity (%)	Specificity (%)	Average Dice coefficient	Minimum Dice coefficient	Maximum Dice coefficient	Accuracy (%)	AUC
Ascites‐1	94.38	68.13	0.65 (SD = 0.21)	<0.01	0.93	81.25	0.76
Ascites‐2	97.12	86.33	0.79 (SD = 0.14)	0.35	0.98	91.73	0.91

AUC, area under the curve; SD, standard deviation.

< 0.01 means that U‐net identifies a wrong region.

## 4 DISCUSSION

Test results showed the U‐net offers high sensitivity but low specificity in the diagnosis of Ascites‐1 US images, with moderate accuracy in the contouring of ascites ROIs. Similarly, the U‐net exhibited both high sensitivity and high specificity in the diagnosis of Ascites‐2, with high accuracy for ascites ROIs division. After reading the output data, we found that image recognition was more accurate for large ascites areas (with large ROIs areas), while the segmentation of lesion areas was consistent. Conversely, image recognition was less accurate for small ascites areas (with small ROIs areas) and more prone to error identification. Ascites areas were relatively small in the Ascites‐1 images and relatively large in the Ascites‐2 images, which may explain why the U‐net performed better for Ascites‐2 than Ascites‐1. In addition, the model parameters selected may not be the best. Although some results predicted by the model are true positive or false positive, the predicted region is wrong. Further study is needed to assess the ability of a U‐net to diagnose and segment small amounts of intraperitoneal fluid. More powerful frameworks like U‐net 3+ may be suitable for Ascites‐1 or Ascites‐3, but this is yet to be investigated.

This study did include some other limitations too, as it utilized specific US images from the hospital and is not necessarily robust to all different equipment versions or manufacturers. Participants were also primarily from the surrounding area and thus represent a limited population sample. Examination parameters were not controlled and varied slightly between individual doctors and treatment environments. In addition, the performance of an AI model is highly dependent on the quantity and quality of training data. Recent developments in US equipment have led to US images exhibiting higher resolution and lower noise. Since the U‐net developed in this study was trained with existing US images, its diagnostic capabilities given higher quality data need to be verified.^52^ Furthermore, our primary goal is to identify the presence of intraperitoneal injuries. However, the ascites US images selected in this study were not all from patients with abdominal trauma, which may have affected trauma recognition accuracy. The effects of fluid volume, intestinal fluid, and peritoneal effusion under normal conditions were not considered. Non‐image information is included in the US images too, which may affect the detection. Some human factors may overestimate the performance of the model, such as the unclear images excluded in the research process. Thus, the differential diagnostic capabilities of the developed U‐net for pathological ascites in US images should be investigated further.

It is not expected that AI should be entirely consistent with the diagnostic capabilities of experienced doctors. Although correctly outlining lesion borders is valuable for treatment planning, determining the presence or absence of abdominal free fluid is the first step in our proposed methodology. The Dice coefficients achieved in this study reflect the difficulty of delineating ascites areas.^58^ The low sensitivity can also be attributed to issues with detecting lesions in abdominal parenchymal organs. Thus, although ultrasound is generally accepted as playing a significant role in the treatment of unstable patients, its use with stable patients is still controversial because it cannot rule out abdominal organ lesions. For patients with blunt trauma, the inability to demonstrate a lack of bleeding or delayed bleeding in the abdominal cavity has proven to be a primary limitation of US, often requiring patients to undergo contrast‐enhanced computed tomography.^21^, ^29^, ^59^ However, as demonstrated in this study, the inclusion of AI could lead to earlier intervention, more precise treatment, and improved outcomes for trauma patients, particularly in emergency situations.

## 5 CONCLUSIONS

The proposed DL‐based methodology has been shown to be accurate for identifying the presence of abdominal free fluid and aiding doctors in diagnosing ascites. Despite the varied resolution of US images, the reported AUC values are comparable to those of recent studies investigating automated diagnosis of edema through varying modalities.^60^ Further clinical validation of the U‐net is needed to demonstrate its effect on patient outcomes, in addition to the reported performance metrics (sensitivities of 94.38% and 97.12% for Ascites‐1 and Ascites‐2, respectively). A robust clinical assessment would require external testing among diverse cohorts that fully represent potential ascites patient groups and images, to avoid performance overestimation caused by overfitting.^56^ In a future study, we will collect US images from additional sources, including various manufacturers, equipment types, scanning parameters, and populations to improve U‐net diagnostic performance. We will also evaluate the potential for differential diagnosis and develop relevant software for use in emergency centers and medical colleges.

## CONFLICT OF INTEREST

The authors declare no conflict of interest.

## ETHICS STATEMENT

This retrospective study was approved by the Clinical Research Ethics Committee of Shenzhen Second People's Hospital.

## AUTHOR CONTRIBUTIONS

Zhanye Lin designed the methodology, acquired the US images, conducted the segmentation, collected patient data, performed the experiments, and wrote the paper. Zhengyi Li proposed the study, guided the methodology and experimental design, and revised the paper. Peng Cao and Yingying Lin provided the U‐net model and assisted in the statistical analysis. Fengting Liang and Libing Huang assisted in the acquisition and segmentation of US images and the collection of patient data. Jiajun He aided in U‐net interpretation and result analysis. All authors read and approved the final manuscript.

## Data Availability

The data that support the findings of this study are available on request from the corresponding author. The data are not publicly available due to privacy or ethical restrictions.
